# Advanced gallbladder inflammation is a risk factor for gallbladder perforation in patients with acute cholecystitis

**DOI:** 10.1186/s13017-018-0169-2

**Published:** 2018-02-20

**Authors:** Stefan Jansen, Maciej Stodolski, Hubert Zirngibl, Daniel Gödde, Peter C. Ambe

**Affiliations:** 1 Department of Surgery, Helios University Hospital Wuppertal, Witten–Herdecke University, Heusnerstr. 40, 42283 Wuppertal, Germany; 2 Department of Pathology and Molecular Pathology, Helios University Hospital Wuppertal, Witten–Herdecke University, Heusnerstr. 40, 42283 Wuppertal, Germany; 3 Department of Visceral, Minimally Invasive and Oncologic Surgery, Marien Hospital Düsseldor, Rochusstr. 2, 40479 Düsseldorf, Germany

**Keywords:** Acute perforated cholecystitis, Gallbladder perforation, Laparoscopic cholecystectomy, Bile duct injury, Morbidity and mortality

## Abstract

**Background:**

Acute perforated cholecystitis (APC) is probably the most severe benign gallbladder pathology with high rates of morbidity and mortality. The cause of APC has not been fully understood. We postulated that APC is a complication of advanced gallbladder inflammation. The aim of this study was to investigate the extent of gallbladder inflammation in patients with APC.

**Methods:**

Patients with intraoperative and histopathologic diagnosis of APC were compared with cases with acute cholecystitis without perforation with respect to the extent of inflammation on histopathology as well as surgical outcomes.

**Results:**

Fifty patients with APC were compared to 150 cases without perforation. Advanced age > 65 years and elevated CRP were confirmed on multivariate analysis as independent risk factors for APC. Advanced gallbladder inflammation was seen significantly more often in patients with APC (84.0 vs. 18.7%). Surgery lasted significantly longer 131.3 ± 55.2 min vs. 100.4 ± 47.9 min; the rates of conversion (22 vs. 4%), morbidity (24 vs. 7%), and mortality (8 vs. 1%) were significantly higher in patients with APC. ICU management following surgery was needed significantly more often in the APC group (56 vs. 15%), and the overall length of stay (11.2 ± 12.0 days vs. 5.8 ± 6.5 days) was significantly longer compared to the group without perforation.

**Conclusion:**

Acute gallbladder perforation in patients with acute cholecystitis represents the most severe complication of cholecystitis. Acute perforated cholecystitis is a sequela of advanced gallbladder inflammation like empyematous and gangrenous cholecystitis and is associated with poor outcome compared to non-perforated cases.

## Background

Acute perforated cholecystitis (APC) defines gallbladder perforation in the setting of acute cholecystitis. This condition is probably one of the most severe benign gallbladder pathology with an associated high risk of morbidity and mortality [1]. Patients with APC generally present with elevated inflammatory markers like white blood count (WBC) and C-reactive protein (CRP) [2, 3]. Besides, advanced age, relevant concomitant medical conditions, and the male sex have been identified as possible patient-dependent risk factors for APC [4–8]. These features, however, have been described in connection with other severe forms of cholecystitis and therefore are not directly indicative of APC [9, 10]. It is therefore non-conclusive whether these clinical factors are secondary to APC per se or herald de facto the presence of advanced gallbladder inflammation. We postulate that APC must be a sequela of severe gallbladder inflammation. The aim of this study, therefore, was to investigate the extent of gallbladder inflammation in patients undergoing emergency or urgent gallbladder surgery and APC.

## Methods

The study protocol was reviewed and approved by the institutional board of review at the Witten/Herdecke, Germany University. A prospectively maintained database of patients undergoing gallbladder surgery in the department of surgery of a university hospital in Germany was reviewed to identify patients undergoing emergency or urgent cholecystectomy for APC between 2013 and 2016. A written consent was received from all patients. The operative notes of identified cases were reviewed to confirm the intraoperative diagnosis of APC and determine the type of perforation. Gallbladder perforation was graded using a modification of Niemeier’s classification [11]. According to this classification, free perforation into the peritoneal cavity is graded as type I, perforation with pericholecystic abscess walled off by adhesions as type II, while a chronic perforation with cholecystoenteric fistula is graded as type III. The surgical specimens of identified cases were re-examined to confirm the diagnosis of APC and determine the extent of inflammation. Patients undergoing emergency or urgent from 2013 to 2016 were randomly chosen from the database to create a control group. The size of the control group was calculated with a 90% power and a 5% alpha assuming that 30% of cases with APC had advanced cholecystitis on histopathology compared to 10% in cases without perforation. The surgical specimens of all patients included in the control group were re-examined by a blinded experienced pathologist, and the extent of inflammation was determined.

Acute cholecystitis in our department is an indication for emergency or urgent surgery. Generally, laparoscopic cholecystectomy (LC) represents the standard of care for acute cholecystitis (AC) in our institution. All emergency or urgent procedures are performed either by an attending surgeon or by a surgical fellow with profound experience in minimal invasive surgery. We perform a four-port LC as described elsewhere [12]. The decision to convert is reached by the attending surgeon. Primary open cholecystectomy is seldom performed in our department. Drains were used as needed. All patients with APC were put on broadband antibiotics following surgery.

Demographic information including age, sex, and body mass index (BMI) were recorded for each patient. Relevant preoperative findings including WBC, CRP, and gallbladder wall thickness measured by abdominal ultrasound as well as the American Society of Anesthesiologists (ASA) score at the time of surgery were documented. Intraoperative data including the duration of surgery defined as time from incision to suture, the type of surgery (LC, conversion from LC to open surgery and primary open surgery), and rate of conversion after attempted LC was noted. The preoperative severity of AC was classified using the previously published severity scoring system by Ambe et al. [13]. Postoperative findings including the need for intensive care unit (ICU) management, the rate of relevant morbidity, and death as well as the length of stay (LOS) defined as the time interval from surgery to discharge were documented.

Blinded hematoxylin and eosin (HE)-stained sections of gallbladder specimens of all patients included in the study were re-examined by an experienced pathologist. Edematous cholecystitis was classified as uncomplicated while gangrenous and empyematous cholecystitis were classified as complicated as described elsewhere [14–16]. To achieve our goal, the extent of gallbladder inflammation following histopathology was compared in both groups.

The data generated was analyzed using the Statistics Package for Social Sciences version 24 (SPSS, IMB Corp, Armonk, NY, USA). The variables were described using absolute number and percentages, while central tendencies were reported using means with the corresponding standard deviations where necessary. Analytic statistics was performed using the chi-square test, Mann-Withney *U* test, or *t* test where necessary. Univariate and multivariate analyses were performed as needed. A 95% confidence interval was used for all calculations. Results were reported as statistically significant whenever *p* < 0.05.

The primary outcome was the extent of gallbladder inflammation defined as complicated vs. uncomplicated. Secondary outcomes included duration of surgery, rate of conversion following attempted LC, rate of relevant morbidity defined as Clavien–Dindo ≥ 3 [17], need for ICU management, rate of mortality, and LOS.

## Results

**Fig. 1:**
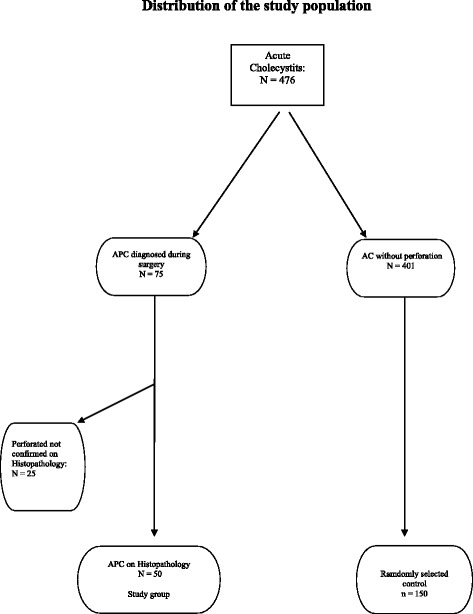
Distribution of the study population. APC: Acute Perforated Cholecystitis

Seventy-five cases of APC were identified based on operative records. Histopathology confirmed APC in 50 cases, which were included in the study group. Type I perforation was seen in one case (2%). Type II perforation was present in 48 cases (96%) including 30 cases (60.0%) with perforation into the large omentum and 18 cases (36%) with perforation towards the liver, while one case (2%) with a cholecysto-duodenal fistula (type III perforation) was recorded. Following sample size analysis as stated above, 150 cases of AC without perforation were included in the control group. Figure 1 shows the distribution of the study population. The demographic characteristics of the study population are presented in Table 1.

**Table 1:** Demographic characteristics of the study population

Features	Study group	Control group	*p* value
Sex			
Female	17 (34.0%)	61 (40.7%)	0.403
Male	33 (66.0%)	9 (59.5%)	
Mean age (years)	73.4 ± 10.0	63.4 ± 16.7	0.001
Mean BMI (kg/m^2^)	28.6 ± 7.2	28.9 ± 13.3	0.870
ASA scores			
1	6 (12.0%)	41 (27.3%)	
2	18 (36.0%)	66 (44.0%)	0.003
3	19 (38.0%)	39 (26.0%)	
> 3	7 (14.0%)	4 (2.7%)	

*ASA* American Society of Anesthesiology score, *BMI* body mass index

The mean WBC was 14.2 ± 6.6/μl in the study group and 12.4 ± 6.3/μl in the control. This difference was not statistically significant, *p* = 0.112. Equally, the mean gallbladder wall thickness did not differ amongst both groups, 6.1 ± 2.6 mm vs. 5.5 ± 2.7 mm, *p* = 0.332. However, the mean CRP was significantly higher in the study group compared to the control group, 19.9 ± 13.5 mg/dl vs. 8.9 ± 10.3 mg/dl, *p* = 0.001.

**Fig. 2:**
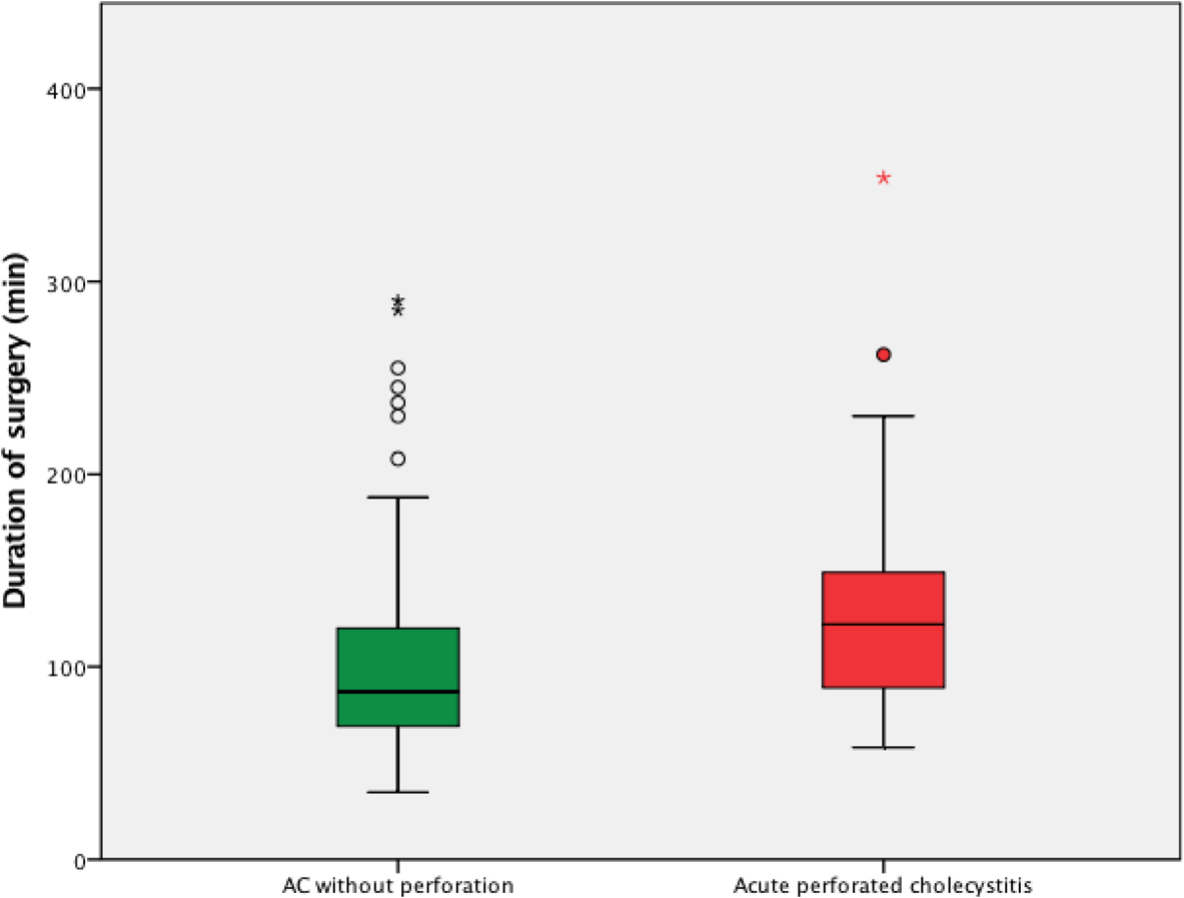
Duration of surgery. Surgery lasted significantly longer in the APC group: 131. 3 ± 55.2 min vs. 100.4 ± 47.9 min, *p* = 0.001

Attempted LC was performed in all cases in the control group, while primary open surgery was performed in four (8%) cases in the study group. Conversion from laparoscopic to open surgery was performed in six cases in the control group (4.0%) compared to 11 cases in the study group (22.0%). This difference was statistically significant, *p* = 0.001. The duration of surgery in both groups is presented in Fig. 2. Surgery lasted significantly longer in the study group.

Postoperative ICU management was needed significantly more often in the study group, 28 (56.0%) cases vs. 23 (15.3%) cases, *p* = 0.001. Grade ≥ 3 complications were recorded in 12 cases (24.0%) in the study group including four (8%) deaths (1× myocardial infarction and 3× sepsis with multi-organ failure), four (8%) cases with bile duct injury, three cases (6%) with intra-abdominal abscess, and one case with surgical site infection. Eleven (7.3%) grade ≥ 3 complications were recorded in the control group including four cases with bile leak (3.0%), two with intra-abdominal abscess, two relevant wound complications, and one case of bleeding, pneumonia, and death (0.7%) following respiratory failure. The rates of morbidity (*p* = 0.003) and mortality (*p* = 0.001) were significantly higher in the APC group. The mean length of hospital stay was significantly longer in the APC group 11.2 ± 12.0 days vs. 5.8 ± 6.5 days, *p* = 0.001.

**Fig. 3:**
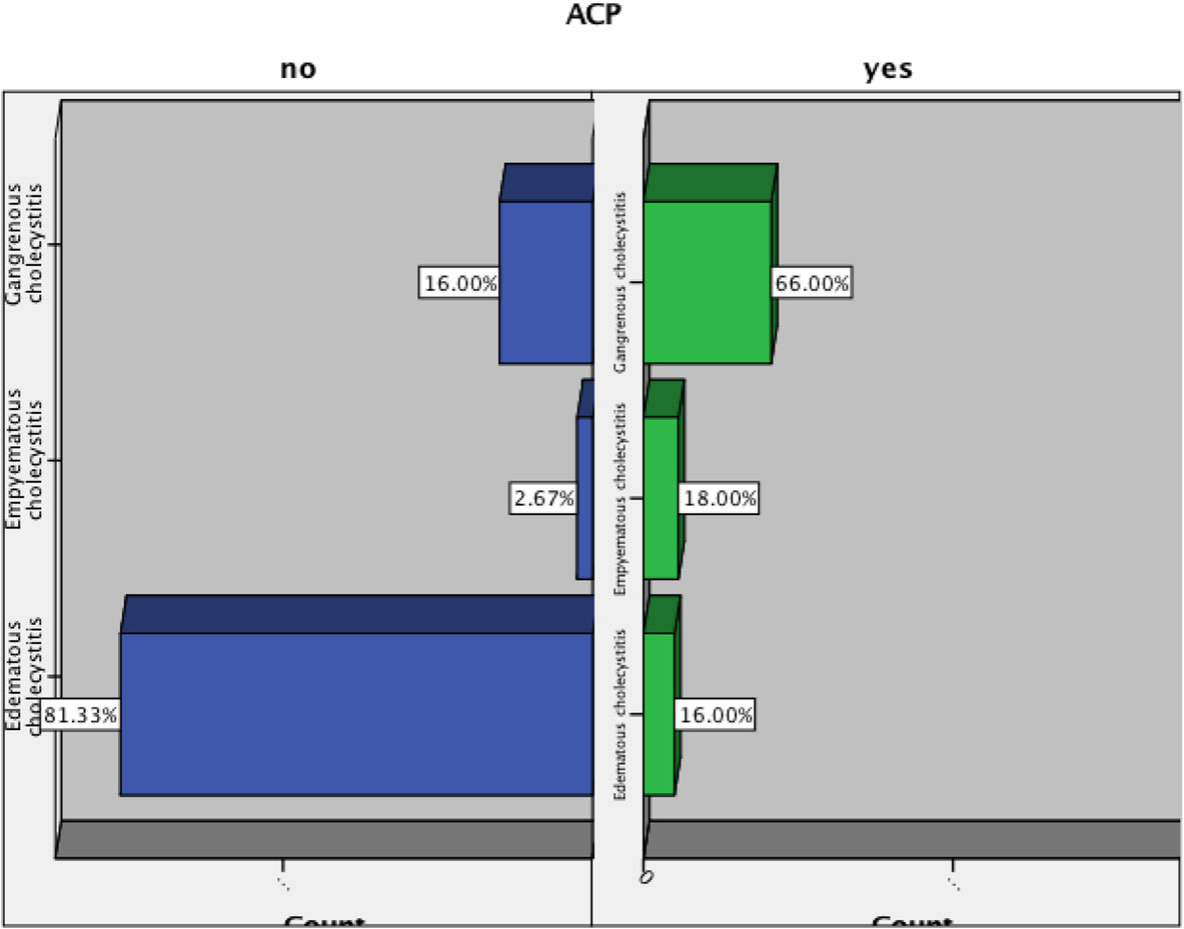
Extent of gallbladder inflammation on histopathology

Histopathology confirmed an advanced form of gallbladder inflammation in the form of empyematous or gangrenous cholecystitis in significantly more cases in the APC group compared to the control group, (84.0 vs. 18.7%, *p* = 0.001), Fig. 3.

## Discussion

This study investigated the extent of gallbladder inflammation in patients undergoing emergency or urgent surgery for APC. Fifty patients with confirmed APC following intraoperative and histological diagnosis were compared to 150 randomly chosen cases undergoing LC for AC without perforation. Advanced gallbladder inflammation with empyematous and gangrenous cholecystitis was recorded significantly more often in the APC group compared to the control. Patients with APC were significantly older and had significantly higher CRP in comparison to the controls. Surgery lasted significantly longer; the rates of conversation, morbidity, and mortality were significantly higher in the APC group. ICU management was needed significantly more often in the APC group, and the overall LOS was significantly longer in the APC group compared to the group without perforation.

Gallbladder perforation in the setting of acute cholecystitis has been suggested in our recently published register study to be a risk factor for poor outcome in patients undergoing cholecystectomy for AC [1]. The outcomes of 5704 cases with APC undergoing emergency or urgent cholecystectomy were compared to those of 39,661 patients without perforation. The duration of surgery, the rates of conversation, morbidity, and mortality were significantly higher in patients with APC compared to those without APC. The results of the present study are in accordance with the results reported in the above study.

A great drawback to the register study by Jansen et al. which was clearly stated by the authors in the limitation section was the inability to characterize the extent of gallbladder inflammation because the register data used in their study did not contain histology findings. This shortcoming was investigated in the present study. Complicated cholecystitis defined as advanced gallbladder inflammation in the form of empyematous or gangrenous cholecystitis was confirmed in a significant portion of the APC group compared to the control group.

Advanced age > 65 years and elevated CRP were identified as risk factors for APC. These factors were further confirmed on multivariate analysis as independent risk factors for APC. These same factors have been previously described in connection with other forms of severe cholecystitis and therefore are not specific for APC [18, 19].

The significantly longer duration of surgery and higher rate of conversion to open surgery following attempted LC in the APC group in comparison to the group without perforation are de facto arguments for the surgical challenge associated with the management of patients with APC. Besides, the 8% rate of bile duct injury and 6% rate of postoperative intra-abdominal abscess formation in the APC group herald the severity of this entity. The mortality rate in this study was 8%. This rate is almost twice the reported rate by Jansen et al. [1] but comparable with the 9.5% rate of death reported in a retrospective analysis of 137 patients with APC by Ausania et al. in 2015 [20]. The heterogeneity in the reported risk of mortality in this and as well as in previous publications must be interpreted in context with the study population. Furthermore, the significantly higher rates of ICU management, as well as the significantly longer LOS, must be interpreted as indicators of the astringent nature of APC.

Another interesting finding in this study was the fact that four patients (8%) in the APC group underwent primary open cholecystectomy compared to none in the control group. These patients underwent explorative laparotomy for acute abdomen with peritonitis, and the diagnosis of APC was reached during surgery. Type I perforation with bilious peritonitis was evident during laparotomy in these patients. This finding should be interpreted as a further demonstration of the severe clinical nature of APC.

The retrospective study design and the small size of the study population must be stated as possible limitations to this study. Thus, further investigations with larger populations would be needed to further investigate the trends shown here. Besides, the results reported in this study might be secondary to the profound expertise in laparoscopic surgery in our department. Therefore, the results of this study might not be readily projected on other institutions. All cases included in this study were managed surgically. Interventional treatment like percutaneous cholecystostomy is not routinely performed in our center. Although the current literature on the role of percutaneous cholecystostomy is not conclusive [21–25], it remains speculative if the outcomes recorded in this study might have been altered by such an interventional management.

Surgeons and clinicians must be aware of the severe nature of APC with associated high risks of morbidity and mortality. The surgical management of such patients warrants profound expertise, and conversion from laparoscopic to open surgery should never be considered a failure. A vigilant postoperative care including the use of broadband antibiotics and close monitoring, e.g., in the ICU, should be considered in such cases.

Taken together, the results of this study confirm APC as a severe complication of AC with significantly higher rates of morbidity and mortality. Advanced gallbladder inflammation including empyematous and gangrenous cholecystitis was seen significantly more often in patients with APC compared to those without perforation. Thus, gallbladder perforation in APC must be secondary to advanced gallbladder inflammation.

## Conclusion

Acute gallbladder perforation in patients with acute cholecystitis represents the most severe complication of cholecystitis. Acute perforated cholecystitis is a sequela of advanced gallbladder inflammation like empyematous and gangrenous cholecystitis and is associated with poor outcome compared to non-perforated cases.

## Abbreviations

AC: Acute cholecystitis
APC: Acute perforated cholecystitis
ASA: American Society of Anesthesiologists
BMI: Body mass index
CRP: C-reactive protein
HE: Hematoxylin and eosin
ICU: Intensive care unit
LC: Laparoscopic cholecystectomy
LOS: Length of stay
WBC: White blood count
